# Supplementary material for: The Automated Test of Embodied Cognition: Concept, Development, and Preliminary Findings
Source: Brain Sci. 2023 May 25;13(6):856. doi: 10.3390/brainsci13060856 (PMC10296682; doi:10.3390/brainsci13060856)
Supplement: Supplementary file 1 [file brainsci-13-00856-s001.zip › brainsci-2370142- File S1.pdf]

Rater:______________________________________ Date:_______________ Subject ID:___________________________

ACTIVATE MOVEMENT CHALLENGE (AMC)

Morris D. Bell, Ph.D.

A. GROSS MOTOR—GAIT AND BALANCE

	RAW	CONVERTED	*SCORING CRITERIA*
1. Timed-Up & Go			> 20 = 0, 20-16=1, <16 = 2
2. Dual Attention			> 20 = 0, 20-16=1, <16 = 2
3. Tandem Gait Forward			Steps correct: <4=0, 4-6 = 1, 7-8 = 2
4. Stand - Arms Outstretched			Seconds: <5 = 0, 5 - 8 = 1, 9 - 10 = 2
5. Stand on One Foot—Right			Seconds: <5 = 0, 5 - 8 = 1, 9 - 10 = 2
6. Stand on One Foot—Left			Seconds: <5 = 0, 5 - 8 = 1, 9 - 10 = 2

A.1 TOTAL GAIT/BALANCE SCORE (ITEM 1 + ITEM2 + ITEM 3)

SCORE 0 - 6 ☐

A.2 TOTAL BALANCE SCORE (ITEM 4 + ITEM 5 + ITEM 6)

SCORE 0 - 6 ☐

B. RHYTHMIC MOVEMENT

**Raw Score = total number of steps in rhythm (step on the beat)*

	RAW	CONVERTED	
1. March to the Beat—Slow			Beats correct: 0-8= 0, 9-16 = 1, 17-20 = 2
2. March to the Beat—Fast			Beats correct: 0-16 = 0, 17-28= 1, 29-32 = 2

B.1 TOTAL RHYTHMIC MOVEMENT SCORE ☐

SCORE 0– 4

Rater:_______________________________ Date:_______________ Subject ID:_____________________________

C. Visuospatial Memory

	ACCURACY	RAW	CONVERTED
1. MapSense3 Trial 1	0-1=0, 2=1, 3=2		
2. MapSense3 Trial 2	0-1=0, 2=1, 3=2		
3. MapSense3 Trial 3	0-1=0, 2=1, 3=2		
4. MapSense4 Trial 1	0-1=0, 2-3=1, 4=2		
5. MapSense4 Trial 2	0-1=0, 2-3=1, 4=2		
6. MapSense4 Trial 3	0-1=0, 2-3=1, 4=2		
7. MapSense5 Trial 1	0-2=0, 3-4=1, 5=2		
8. MapSense5 Trial 2	0-2=0, 3-4=1, 5=2		
9. MapSense5 Trial 3	0-2=0, 3-4=1, 5=2		

RHYTHM/COORDINATION

		1
3	2	

Key	RAW	CONVERTED
0-1=0, 2=1, 3=2		
0-1=0, 2=1, 3=2		

1		
	2	
	3	4

Key	RAW	CONVERTED
0-1=0, 2-3=1, 4=2		
0-1=0, 2-3=1, 4=2		

	1	
	2	3
5	4	

Key	RAW	CONVERTED
0-2=0, 3-4=1, 5=2		
**0-2=0, 3-4=1, 5=2**		

C.1 TOTAL VISUOSPATIAL MEMORY ACCURACY SCORE

SCORE 0 - 18 ☐

C.2 TOTAL VISUOSPATIAL MEMORY RHYTHM/ COORDINATION SCORE

SCORE 0 - 12 ☐

D. BILATERAL COORDINATION

each pass get up two rhythm points; one for each beat

	ACCURACY RAW	ACCURACY CONVERTED	RHYTHM RAW	RHYTHM CONVERTED	
1. Bi-manual Ball Pass—Slow					Passes correct: 0 - 4 = 0, 5– 6 = 1, 7– 8 = 2
2. Bi-manual Ball Pass—Fast					Rhythm: 0 - 8 = 0, 9– 12 = 1, 13– 16 = 2

D.1 TOTAL BILATERAL COORDINATION

ACCURACY SCORE 0– 4 ☐

D.2 TOTAL BILATERAL COORDINATION

RHYTHM SCORE 0– 4 ☐

Rater:______________________________________ Date:________________ Subject ID:____________________________

E. AUDITORY UPPER BODY ACCURACY, RHYTHM AND RESPONSE INHIBITION

**0: moves during both beats, 1: moves only on first, 2: still*

Green Lights correct: 0 - 5= 0, 6– 8= 1, 9– 10= 2	Red Lights correct: 0 - 6 = 0, 7-10= 1, 11-12= 2	Green Lights Rhythm: 0 - 10 = 0, 11-16= 1, 17- 20= 2

Bi-manual Ball Pass Red/Green Light

	GREEN LIGHTS CORRECT		RED LIGHTS CORRECT		GREEN RHYTHM CORRECT	
	RAW	CONVERTED	RAW	CONVERTED	RAW	CONVERTED
1. Auditory—Slow	☐	☐	☐	☐	☐	☐
2. Auditory —Fast	☐	☐	☐	☐	☐	☐

Green/Yellow Lights correct: 0 - 6= 0, 7– 10= 1, 11– 12= 2	Red Lights correct: 0 - 4 = 0, 5-6= 1, 7-8= 2	Green/Yellow Lights Rhythm: 0 - 12 = 0, 13-20= 1, 21-24= 2

Ball Pass Red/Green/Yellow Light

	GREEN/YELLOW LIGHTS CORRECT		RED LIGHTS CORRECT		G/Y RHYTHM CORRECT	
3. Auditory—Slow	☐	☐	☐	☐	☐	☐
4. Auditory —Fast	☐	☐	☐	☐	☐	☐

E.1 TOTAL AUDITORY ACCURACY

SCORE 0 - 8 ☐

E.2 TOTAL AUDITORY RESPONSE INHIBITION

SCORE 0 - 8 ☐

E.3 TOTAL AUDITORY RHYTHM

SCORE 0 - 8 ☐

E.4 TOTAL G/Y LIGHT ACCURACY

SCORE 0 - 4 ☐

E.5 TOTAL RED LIGHT ACCURACY

SCORE 0 - 4 ☐

F. VISUAL UPPER BODY ACCURACY, RHYTHM AND RESPONSE INHIBITION

*** No beats (rhythm)*

0: movement during the whole video

1: self-correct (move and then keep still)

2: no movement — still

Green/Yellow Lights correct: 0 - 6= 0, 7– 10= 1, 11– 12= 2	Red Lights correct: 0 - 4 = 0, 5-6= 1, 7-8= 2

Bag Pass Red/Green/Yellow Light

	GREEN/YELLOW LIGHTS CORRECT		RED LIGHTS CORRECT	
1. Visual—Slow	☐	☐	☐	☐
2. Visual —Fast	☐	☐	☐	☐

F.1 TOTAL VISUAL ACCURACY

SCORE 0 - 4 ☐

F.2 TOTAL VISUAL RESPONSE INHIBITION

SCORE 0 - 4 ☐

Rater:______________________________________ Date:_______________ Subject ID:___________________________

G. BI-LATERAL COORDINATION AND SELF-REGULATION

**Accuracy Raw Score = total number of body parts touched at least once*

**Rhythm/Coordination Raw Score = total number of times correctly touching body part with the opposite hand all three times, accurately keeping the beat for all three touches. Starting late and rushing to catch the beat is not correct*

	Accuracy scale	Accuracy RAW SCORE	Accuracy CONVERTED	Rhythm/Coordination scale	Rhythm/Coordination RAW SCORE	Rhythm/Coordination CONVERTED
1. Trial 1—Ears, Shoulders, Hips and Knees<br>***If Converted Accuracy score = 0, go to H.***	0-1=0, 2-3=1, 4=2			0-6=0, 7-10=1, 11-12=2		
2. Trial 2—Opposite Ears and Knees<br>***If Converted Accuracy score = 0, go to H.***	0-4=0, 5-7=1, 8=2			0-12=0, 13-20=1, 21-24=2		
3. Trial 3—Opposite Hips and Shoulders<br>***If Converted Accuracy score = 0, go to H.***	0-4=0, 5-7=1, 8=2			0-12=0, 13-20=1, 21-24=2		
4. Trial 4—Opposite Ears, Knees, Hips and Shoulders	0-6=0, 7-10=1, 11-12=2			0-18=0, 19-32=1, 33-36=2		

G.1 TOTAL ACCURACY SCORE

SCORE 0 - 8 ☐

G.2 TOTAL RHYTHM/COORDINATION SCORE 0 - 8 ☐

G.3 WORKING MEMORY ACCURACY SCORE (#2—#4)

SCORE 0 - 6 ☐

H. Embodied Memory Recall

	ACCURACY scale	ACCURACY RAW	ACCURACY CONVERTED	Pattern	RHYTHM/COORDINATION scale	RHYTHM/COORDINATION RAW	RHYTHM/COORDINATION CONVERTED
1. MapSense3 Recall	0-1=0, 2=1, 3=2			see grid 1	0-1=0, 2=1, 3=2		
2. MapSense4 Recall	0-1=0, 2-3=1, 4=2			see grid 2	0-1=0, 2-3=1, 4=2		
3. MapSense5 Recall	0-2=0, 3-4=1, 5=2			see grid 3	0-2=0, 3-4=1, 5=2		

		1
3	2	

1		
	2	
	3	4

	1	
	2	3
5	4	

H.1 TOTAL VISUOSPATIAL MEMORY RECALL ACCURACY SCORE

SCORE 0 - 6 ☐

H.2 TOTAL VISUOSPATIAL MEMORY RECALL RHYTHM/COORDINATION SCORE

SCORE 0 - 6 ☐

I. RAPID/SEQUENTIAL MOVEMENTS SCORING CRITERIA

1. FOOT TAP -Rapidity Raw score = total number of correct foot taps in 10 seconds.
0-9 =0; 10-19 = 1; >20 = 2

2. FOOT STOMP- Rapidity Raw score = total number of correct foot stomp movements in 10 seconds.
0-9 =0; 10-19 = 1; >20 = 2

3. FIST OPEN CLOSE -Rapidity Raw score = total number of correct fist opening times in 10 seconds.
0-9 =0; 10-19 = 1; >20 = 2

4. HAND PRONATE/SUPINATE -Rapidity Raw score = total number of correct Pronate/Supinate movements in 10 seconds. 0-5 =0; 6-9 = 1; >10 = 2

5. FINGER TAP -Rapidity Raw score = total number of correct Finger taps in 10 seconds.
0-15 =0; 16-29 = 1; >30 = 2

Fluidity Score ALL TASKS — Smoothness and accuracy of motion (freezes up or other movements intrude).

Stops and doesn't restart or switches to another movement and doesn't change back to the correct movement = 0.

Stops but restarts, has intrusive movements but returns to correct movements or has mirror movements = 1.

Continuous correct movement = 2.

H. RAPID/SEQUENTIAL MOVEMENTS

FLUIDITY CONVERTED SCORE

RAPIDITY CONVERTED SCORE

RAPIDITY RAW SCORE

	RAPIDITY RAW SCORE	RAPIDITY CONVERTED SCORE		FLUIDITY CONVERTED SCORE
FOOT TAP—RIGHT FOOT (10 SEC)				
FOOT TAP—LEFT FOOT (10 SEC)				
FOOT STOMP—RIGHT FOOT (10 SEC)				
FOOT STOMP—LEFT FOOT (10 SEC)				
FIST OPEN CLOSE —RIGHT HAND (10 SEC)				
FIST OPEN CLOSE —LEFT HAND (10 SEC)				
HAND PRONATE/SUPINATE—RIGHT HAND (10 SEC)				
HAND PRONATE/SUPINATE—LEFT HAND (10 SEC)				
FINGER TAP—RIGHT HAND (10 SEC)				
FINGER TAP—LEFT HAND (10 SEC)				
TOTAL SCORE				

I.1 TOTAL RAPIDITY SCORE

I.2 TOTAL FLUIDITY SCORE

Rater:______________________________ Date:______________ Subject ID:________________________

SUMMARY OF SCORES

BALANCE

Gait/Balance Total (A.1) ☐ + Balance Total (A.2) ☐ = Balance Total ☐

0-1 = 0; 2-3 = 1, 4-5 = 2; 6-8 = 3; 9-10 = 4; 11-12 = 5

BALANCE CONVERTED SCORE ☐

WORKING MEMORY

Yellow Light Accuracy Total (E.1+F.1) ☐ + Cross Body Opposites (G.3) ☐ + MapSense (C.1) ☐ = Working Memory Total ☐

0-6 = 0; 7-13 = 1; 14-21 = 2; 22-27 = 3; 28-32 = 4; 33-36 = 5

WORKING MEMORY CONVERTED SCORE ☐

RESPONSE INHIBITION

Bag Pass Response Inhibition Total (E.2+F.2) ☐ + Cross Body Rhythm Total (G.2) ☐ = Response Inhibition Total ☐

0-3 = 0; 4-6 = 1, 7-10 = 2; 11-14 = 3; 15-17 = 4; 18-20= 5

RESPONSE INHIBITION CONVERTED SCORE ☐

SELF-REGULATION

Cross Body Accuracy (G.1) ☐ + Cross Body Rhythm Total (G.2) ☐ = Self-Regulation Total ☐

0-1 = 0; 2-3 = 1, 4-8 = 2; 9-12 = 3; 13-14 = 4; 15-16 = 5

SELF-REGULATION CONVERTED SCORE ☐

Rhythm/Coordination

Upper Body Rhythm Total (D.2+E.3) ☐ + Whole Body Rhythm Total (G.2) ☐ + MapSense (C.2) ☐ + MapSense Recall Rhthym/Coordination (H.2) ☐ = Rhythm/ Coordination Total ☐

0-5 = 0; 6-12 = 1; 13-18 = 2; 19-25 = 3; 26-32 = 4; 33-38 = 5

RHYTHM/COORDINATION CONVERTED SCORE ☐

Rater:______________________________________ Date:_______________ Subject ID:____________________________

ATTENTION

Bag Pass Accuracy Total (D.1+E.1+F.1) [ ] + Cross Body Accuracy Total (G.1) [ ] + MapSense (C.1) [ ] = Attention Total [ ]

0-5 = 0; 6-13 = 1; 14-20 = 2; 21-30 = 3; 31-37 = 4; 38-42 = 5

ATTENTION CONVERTED SCORE [ ]

MOTOR SPEED

Rapidity Total (I.1) [ ] + Fluidity Total (I.2) [ ] = Motor Speed Total [ ]

0-5 = 0; 6-13 = 1; 14-20 = 2; 21-30 = 3; 31-36 = 4; 37-40 = 5

MOTOR SPEED CONVERTED SCORE [ ]

EMBODIED MEMORY RECALL

Visual Memory Recall Accuracy (H.1) [ ] = Visual Memory Recall Total [ ]

0= 0; 1= 1; 2 = 2; 3-4 = 3; 5 = 4; 6 = 5

VISUAL MEMORY CONVERTED SCORE [ ]

Rater:______________________________ Date:______________ Subject ID:______________________

AMC TOTAL SCORE

BALANCE CONVERTED SCORE	☐
	+
WORKING MEMORY CONVERTED SCORE	☐
	+
RESPONSE INHBITION CONVERTED SCORE	☐
	+
SELF-REGULATION CONVERTED SCORE	☐
	+
RHYTHM/COORDINATION CONVERTED SCORE	☐
	+
ATTENTION CONVERTED SCORE	☐
	+
EMBODIED MEMORY RECALL CONVERTED SCORE	☐
	+
MOTOR SPEED CONVERTED SCORE	☐
	=
**AMC TOTAL SCORE**	☐

Performance Categories

**Very Severely Impaired**	**0—15**	☐
**Severely Impaired**	**16—18**	☐
**Moderately Severely Impaired**	**19—22**	☐
**Moderately Impaired**	**23—27**	☐
**Mildly Impaired**	**28—32**	☐
**Minor Difficulties**	**33—36**	☐
**No Difficulties**	**37—40**	☐

Rater:______________________________________ Date:_______________ Subject ID:___________________________

SUPPLEMENTARY SCORES

Discrepancy - Right / Left

Right	Left
A.5	A.6
H.1	H.2
H.3	H.4
H.5	H.6
H.7	H.8
H.9	H.10
Total =	Total =

☐ − ☐ = ☐

Discrepancy - Auditory / Visual

Auditory	Visual
E.4	F.1
E.5	F.2
Total =	Total =

☐ − ☐ = ☐

Discrepancy

Timed Up & Go / Dual Attention

☐ − ☐ = ☐

Discrepancy - Accuracy / Response Inhibition

Accuracy	Response Inhibition
E.1	E.2
F.1	F.2
Total =	Total =

☐ − ☐ = ☐

Participant Experience Questionnaire
ACTIVATE TEST OF EMBODIED COGNITION

ID Number ______________________________ Date ________________________________

Please rate the following statements about the ATEC assessment experience using a scale of 1 to 5, where **1 indicates that you STRONGLY DISAGREE and 5 indicates you STRONGLY AGREE**.

	Strongly Disagree				Strongly Agree
1. I found that some of the tasks were challenging.	1	2	3	4	5
2. I was often bored during the assessment.	1	2	3	4	5
3. I liked the video host, and thought the demonstrations were helpful.	1	2	3	4	5
4. I liked doing some tasks I had never done before.	1	2	3	4	5
5. I always felt safe doing the tasks.	1	2	3	4	5
6. I thought the assessment was relevant to my medical and physical health.	1	2	3	4	5
7. I would not hesitate to take this assessment again.	1	2	3	4	5
8. Instructions were confusing, and I usually didn't understand what I was supposed to do.	1	2	3	4	5
9. Overall, I felt comfortable with the assessment process.	1	2	3	4	5
10. Overall, I enjoyed doing the tasks.	1	2	3	4	5

Semi Structured Interview for Qualitative Assessment

Did you feel this assessment was enjoyable*?* Yes No
Why or why not?

What task(s) were your favorite? Why?

What task(s) did you dislike? Why?

Did you feel safe and comfortable during the assessment*?* Yes No
Why or Why not?

What task(s) if any made you feel unsafe? Why?

If you found some tasks uncomfortable, what could we do to make it better for you?

Do you think that this assessment is relevant for understanding your mental or physical health problems? Yes No

Why or Why not?

Would you be willing to take this assessment again? Yes No

Why or Why not?

Is there anything else you would like to tell us about your experience taking this assessment?

Thank you for sharing your experience and for participating in this process.
