# Supplementary material for: The Automated Test of Embodied Cognition: Concept, Development, and Preliminary Findings
Source: Brain Sci. 2023 May 25;13(6):856. doi: 10.3390/brainsci13060856 (PMC10296682; doi:10.3390/brainsci13060856)
Supplement: Supplementary file 1 [file brainsci-13-00856-s001.zip › brainsci-2370142-File S2.pdf]

Participant Experience Questionnaire
ACTIVATE TEST OF EMBODIED COGNITION

ID Number ______________________________ Date ________________________________

Please rate the following statements about the ATEC assessment experience using a scale of 1 to 5, where **1 indicates that you STRONGLY DISAGREE and 5 indicates you STRONGLY AGREE**.

	Strongly Disagree				Strongly Agree
1. I found that some of the tasks were challenging.	1	2	3	4	5
2. I was often bored during the assessment.	1	2	3	4	5
3. I liked the video host, and thought the demonstrations were helpful.	1	2	3	4	5
4. I liked doing some tasks I had never done before.	1	2	3	4	5
5. I always felt safe doing the tasks.	1	2	3	4	5
6. I thought the assessment was relevant to my medical and physical health.	1	2	3	4	5
7. I would not hesitate to take this assessment again.	1	2	3	4	5
8. Instructions were confusing, and I usually didn't understand what I was supposed to do.	1	2	3	4	5
9. Overall, I felt comfortable with the assessment process.	1	2	3	4	5
10. Overall, I enjoyed doing the tasks.	1	2	3	4	5

Semi Structured Interview for Qualitative Assessment

Did you feel this assessment was enjoyable*?* Yes No
Why or why not?

What task(s) were your favorite? Why?

What task(s) did you dislike? Why?

Did you feel safe and comfortable during the assessment*?* Yes No
Why or Why not?

What task(s) if any made you feel unsafe? Why?

If you found some tasks uncomfortable, what could we do to make it better for you?

Do you think that this assessment is relevant for understanding your mental or physical health problems? Yes No

Why or Why not?

Would you be willing to take this assessment again? Yes No

Why or Why not?

Is there anything else you would like to tell us about your experience taking this assessment?

Thank you for sharing your experience and for participating in this process.
